# Nasopharyngeal carriage of *Streptococcus pneumoniae* among children aged 30 days to <60 months in Beijing and Shenzhen, China (2018–2021) during pneumococcal conjugate vaccine introduction and the coronavirus disease (COVID-19) pandemic

**DOI:** 10.3389/fped.2024.1382165

**Published:** 2024-09-03

**Authors:** Qianqian Du, Zhaoqiu Liu, Hongmei Wang, Yani Wang, Li Liu, Xuexia Wen, Sangjie Yu, Qingqing Ren, Elisa Gonzalez, Adriano Arguedas, Mark A. Fletcher, Kaijie Pan, Graciela Del Carmen Morales, Jikui Deng, Kaihu Yao

**Affiliations:** ^1^ Key Laboratory of Major Diseases in Children, Ministry of Education, National Clinical Research Center for Respiratory Diseases, National Key Discipline of Pediatrics, Laboratory of Infection and Microbiology, Beijing Pediatric Research Institute, Beijing Children’s Hospital, Capital Medical University, National Center for Children’s Health, Beijing, China; ^2^ Department of Preventive Health, First Affiliated Hospital of Tsinghua University, Beijing, China; ^3^ Division of Infectious Diseases, Shenzhen Children’s Hospital, Shenzhen, Guangdong, China; ^4^ Shenzhen Nanshan Medical Group Headquarters, Taohua Yuan Community Health Service Center, Shenzhen, Guangdong, China; ^5^ Department of Preventive Health, Beijing Children’s Hospital, Capital Medical University, Beijing, China; ^6^ Shenzhen Nanshan Medical Group Headquarters, Chiwan Community Health Service Center, Shenzhen, Guangdong, China; ^7^ Shenzhen Nanshan Medical Group Headquarters, Haiwan Community Health Service Center, Shenzhen, Guangdong, China; ^8^ Vaccines/Antivirals & Evidence Generation, Pfizer, Inc., Collegeville, PA, United States; ^9^ Vaccines & Antivirals, Emerging Markets, Pfizer, Inc., Paris, France; ^10^ Vaccines, Medical Affairs, Emerging Markets, Pfizer, Inc., New York, NY, United States

**Keywords:** *Streptococcus pneumoniae*, nasopharyngeal carriage, pneumococcal conjugate vaccine, serotypes, antimicrobial resistance

## Abstract

**Objectives:**

To describe the carriage rate, serotype distribution, and antimicrobial susceptibility patterns of *Streptococcus pneumoniae* (*S. pneumoniae*) nasopharyngeal (NP) isolates among healthy children aged 30 days to <60 months in the cities of Beijing and Shenzhen during 2018–2021.

**Methods:**

A NP swab sample was collected among four annual cohorts of healthy children at routine well-child visits. *S. pneumoniae* was identified by culture, optochin sensitivity and bile solubility, serotypes determined by latex agglutination and Quellung, and antimicrobial susceptibility testing performed using E-test strips.

**Results:**

*S. pneumoniae* NP carriage was 13.1% (645/4,911), with the highest *S. pneumoniae* carriage prevalence (15.3%) observed in 25 to <60 months. The carriage prevalence was 15.1% in children 13–24 months, 13.2% in children 7–12 months, and 8.2% in children 30 days to 6 months (*P* < 0.01). Living with siblings [20.0% vs. 9.4%: OR: 2.42 (95% CI: 2.05–2.87)] or attending day-care [31.8% vs. 11.3%: OR: 3.67 (95% CI: 2.94–4.57)] increased the risk (*P* < 0.01). During the period (January 2020–April 2021) of strict non-pharmaceutical interventions to prevent and control the COVID-19 pandemic, the proportion of children with *S. pneumoniae* colonization declined from 16.0% (94/587) to 5.8% (108/1,848) in Beijing while increasing from 14.5% (64/443) to 18.6% (379/2,033) in Shenzhen. Among *S. pneumoniae* isolates, 36.7% (237/645) belonged to 13-valent pneumococcal conjugate vaccine (PCV13) serotypes, 64.3% (408/645) were non-PCV13 serotypes, including 20.8% (134/645) non-serotypeable *S. pneumoniae* (NST). A total of 158/644 isolates (24.5%) were MDR. For the PCV13 isolates, MDR was detected in 36.3% (86/237) of isolates; in comparison, 17.6% (72/407) of non-PCV13 serotypes, including NST, were MDR (*P* < 0.01). *S. pneumoniae* NP carriage was detected in 10.7% of children with previous pneumococcal vaccination (PCV7 or PCV13 only) compared with 14.9% in children without previous pneumococcal vaccination.

**Conclusions:**

The highest *S. pneumoniae* carriage prevalence were found in the oldest age group (25 to <60 months) and in children living with siblings or attending day-care. Vaccination with PCV7 or PCV13 was associated with lower PCV13-serotype colonization. In Beijing, *S. pneumoniae* carriage significantly declined during the COVID-19 pandemic.

## 1 Introduction

*Streptococcus pneumoniae* (*S. pneumoniae*), which can cause invasive and non-invasive diseases, is the main bacterial pathogen for community–acquired pneumonia (CAP) and mucosal diseases such as otitis media (1). *S. pneumoniae* frequently colonizes the human nasopharynx and is transmitted through respiratory droplets. The cross-sectional point prevalence of nasopharyngeal (NP) carriage in infants and young children, who are the main reservoir of this organism, ranges from 27% to 85% (2). NP carriage rates are particularly high among children in low- and middle-income countries (LMICs), as well as among children in high*-*income countries who belong to indigenous populations (1, 3). In China, there are limited data regarding *S. pneumoniae* carriage in healthy children; however, previous studies, based on pediatric inpatients with pneumococcal infections, indicated geographical differences in serotype distribution between northern and southern China (4). Therefore, the present cross-sectional study in China, designed to investigate the *S. pneumoniae* NP carriage rate, serotype distribution, and antimicrobial susceptibility patterns among healthy children aged 30 days to <60 months, involved one northern city and one southern city.

Until the 13-valent pneumococcal conjugate vaccine (PCV13) licensure in China in October 2016, PCV7 was available in the private market for children less than 5 years of age, although uptake was <10% (5). Likewise, PCV13 has been used in the private market, and it is not included in the national immunization program (NIP). PCV13 administration in children in China is based on the principle of “informed consent and voluntary payment”: from 2016, it was only for children less than 15 months of age (6); after 2023, the indication was expanded to the age of 5 years (7). Recent data show that the vaccination rate of PCV13 was increasing year by year in economically developed cities. For example, in Huangpu District in Shanghai, a study reported that the PCV13-Pfizer (full series) uptake increased from 2016 to 2019, ranging from 1.9% (2016), 15.6% (2017), 33.3% (2018), and 42.7% (2019) (8).

Currently, there are 21.5 million people living in Beijing, of whom about 13.5 million are classified as “local residents.” The annual birth cohort is approximately 170 thousand (9). In Beijing, the uptake of non-national immunization program (non-NIP) vaccines in the private sector, including PCV, varies significantly between years and districts. Data from one survey showed that non-NIP vaccine uptake ranged from 3.2% (influenza) to 95.8% (varicella) among children born between 2001 and 2006 (10). Another survey reported that the uptake of PCV7 in Beijing was approximately 18% among children born between 2011 and 2013 (11).

Shenzhen is a developed city with a population of 11 million, of whom 3.5 million are “local residents.” The annual birth cohort is approximately 71 thousand (12). The uptake for non-NIP vaccines is greater in Shenzhen than in other cities. For example, within one Shenzhen district, pneumococcal vaccine uptake [including PCV7 or the 23-valent pneumococcal polysaccharide vaccine (PPSV23)] among children younger than 7 years of age was estimated at 51% (13).

The present study was performed from January 2018 to November 2021, which covered the early stage of PCV13 availability for children <15 months of age, and the study extended into the first 2 years of the COVID-19 pandemic. From February 2020, measures were enforced which included mandatory self-isolation for individuals presenting with respiratory symptoms or fever, requirements for physical distancing maintaining a separation of over 1.5 m, and restrictions limiting the size of social gatherings.

## 2 Methods

### 2.1 Study design

This was a cross-sectional study designed to collect NP swabs among children aged 1–60 months living in Beijing (included two enrolling sites: Beijing Children's Hospital affiliated with Capital Medical University and the First Affiliated Hospital of Tsinghua University) and Shenzhen (included four enrolling sites: Shenzhen Children's Hospital and three Community Health Service Centers). The planned number of enrolled children was close to 5,000. Enrolled children were stratified into four age groups: 30 days to 6 months, 7–12 months, 13–24 months, and 25 to less than 60 months. The planned distribution aimed for approximately 25% of participants in each age group.

### 2.2 Enrollment and study procedures

Children aged 30 days to <60 months who resided in Beijing or Shenzhen were enrolled if determined to be healthy by medical history and the judgment of the investigator. Children with a major congenital malformation or serious chronic disorder were excluded, as were children who participated previously in this study. Children with any of the following conditions were temporarily excluded: (1) current upper or lower respiratory illness or a febrile episode (axillary temperature of ≥38.0°C) within the last 24 h; (2) using antibiotics within the previous 10 days; or (3) history of hospitalization or medical consultation for any type of illness within the previous 15 days. Informed consent was signed by the parents or legal guardians prior to any research procedures. A brief medical history was done, and details were obtained on demographics, previous antimicrobial use within the previous 10–30 days, possible risk factors (i.e., living in a residence with a smoker, living with one or more siblings, attending day-care, parent/guardian working in a medical institution, etc.), and the pneumococcal vaccination history (via the “Child Immunization Record Booklet”). Children with an incomplete, missing, or unclear “Child Immunization Record Booklet” were classified as having an “uncertain vaccination” status. During this visit, a single NP swab was collected by a medically qualified health care professional for the detection of *S. pneumoniae*.

### 2.3 Laboratory methods

NP swabs were taken using the World Health Organization (WHO)-recommended methodology. The specimens were processed based on the WHO recommendations for characterizing *S. pneumoniae* (14). Broth enrichment for 4 h was an approach used to increase the sensitivity of cultures (15). On the same day as swabbing, NP samples were inoculated onto Columbia agar (with 5% sheep blood and 5.0 μg/ml gentamicin) and were incubated aerobically at 37°C in 5% CO_2_ for 48 h. The suspected strains were identified using the optochin sensitivity (>14 mm with a 6-mm optochin disk incubated overnight in 5% CO2) and bile lysis tests. Strains that were positive for both tests were identified as *S. pneumoniae* and included in the study.

Serogroups were tested using the Pneumotest-Latex kit (Statens Serum Institute, Copenhagen, Denmark), and serotypes were determined by the Quellung reactions using factor antisera (Statens Serum Institute, Copenhagen, Denmark) as previously described (16). Non-serotypeable *S. pneumoniae* was defined as an optochin and bile solubility positive isolate with no reaction detectable under the microscope both to the Pneumotest-Latex kit for serogroup and to the Omni antiserum for serotype (Statens Serum Institute, Copenhagen, Denmark). Minimum inhibitory concentration (MIC) values to amoxicillin, penicillin (meningitis and non–meningitis), ceftriaxone, erythromycin, imipenem, meropenem, levofloxacin, linezolid, vancomycin, and sulfamethoxazole/trimethoprim were obtained by means of the E-test strips (PDM Epsilometer, AB Biodisk, Solma, Sweden) following Clinical and Laboratory Standards Institute 2019 criteria (17). Penicillin was interpreted according to the criteria for meningitis (intravenous) and non-meningitis (intravenous or oral) (18). Ceftriaxone was interpreted according to non–meningitis. *S. pneumoniae* American Type Culture Collection 49619 (ATCC49619) was used as the quality control strain and included in each set of tests. Multi-drug resistant (MDR) *S. pneumoniae* were defined as being resistant to three or more antimicrobials classes evaluated in this study. For the Penicillin-I class, the penicillin (oral) resistance rate was used to calculate the MDR.

### 2.4 Statistical analysis

Descriptive statistics were used to summarize the subjects' baseline characteristics. The confidence intervals (CIs) for the proportions were computed using the F distribution as described in the Collett method (19) and implemented in SAS PROC FREQ. Odds ratios (OR) and 95% CIs with the Wald method (20) were used to compare the carriage prevalence of *S. pneumoniae* and of PCV13 serotypes between age groups. The *χ*^2^ test and Fisher's exact test were used for significance comparison between groups via SPSS 26.0 (SPSS Inc., Cary, NC). *P* < 0.05 was deemed to indicate statistical significance.

## 3 Results

### 3.1 Characteristics of the study population

In total, 4,911 healthy children were involved in this analysis (Supplementary Figure 1), evenly distributed across the four age groups, which included 1,103 (22.5%) children aged 30 days to 6 months, 1,184 (24.1%) children aged 7–12 months, 1,240 (25.2%) children aged 13–24 months, and 1,384 (28.2%) children aged 25 to <60 months. Male subjects accounted for 54.6% (*n* = 2,683) of the enrolled children. The vaccination information of study participants is shown in Supplementary Table 1. There were 3 and 166 children previously vaccinated with 3 doses of PCV7 or a single dose of PPSV23, respectively. Among study participants, the proportion of children vaccinated with PCV13 in Shenzhen was 8.4% (208/2,476, 1 dose PCV13), 8.4% (208/2,476, 2 doses PCV13) and 20.4% (505/2,476, 3 doses PCV13), respectively, compared with 4.3% (105/2,435, 1 dose of PCV13), 4.0% (98/2,476, 2 doses of PCV13) and 16.3% (396/2,476, 3 doses of PCV13) in Beijing. Moreover, the proportion of children who completed 4 doses of PCV13 vaccination in Beijing was 13.2%, and it was 12.8% in Shenzhen.

### 3.2 Carriage rate and risk factors

The overall *S. pneumoniae* carriage prevalence was 13.1% (*n* = 645), with different carriage rates between Beijing and Shenzhen (8.3% vs. 17.9%, respectively) (*P* < 0.01). Carriage prevalence increased by age group. Overall, a 15.3% *S. pneumoniae* carriage prevalence was found in the oldest age group of 25 to <60 months, while the carriage prevalence was 15.1% in children 13–24 months, 13.2% in children 7–12 months, and 8.2% in children 30 days to 6 months (*P* < 0.01, Table 1). This pattern of increasing *S. pneumoniae* carriage prevalence with age was observed in both Beijing and Shenzhen. In Beijing, it was 5.6% in children aged 30 days to 6 months, 6.4% in children aged 7–12 months, 9.6% in children aged 13–24 months, and 10.7% in children aged 25 to <60 months; in Shenzhen, it was 11.3%, 18.1%, 19.2%, and 21.6%, respectively.

**Table 1:** Epidemiological factors associated with pneumococcal carriage in children aged 30 days to <60 months.

	Positive no./n	Proportion with carriage (%)	OR (95% CI)^a^	*P* value
Age				<0.01
30 days to 6 months	90/1,103	8.2	0.49 (0.38, 0.64)	
7–12 months	156/1,184	13.2	0.84 (0.67, 1.05)	
13–24 months	187/1,240	15.1	0.98 (0.79, 1.22)	
25 to <60 months	212/1,384	15.3	1	
Gender				0.16
Female	276/2,227	12.4	0.89 (0.75, 1.05)	
Male	369/2,683	13.8	1	
Year enrolled				<0.01
2018	16/131	12.2	0.84 (0.49, 1.43)	
2019	142/899	15.8	1.13 (0.91, 1.40)	
2020	193/1,820	10.6	0.71 (0.59, 0.87)	
2021	294/2,061	14.3	1	
City				<0.01
Beijing	202/2,435	8.3	0.42 (0.35, 0.50)	
Shenzhen	443/2,476	17.9	1	
Received antimicrobial within the past 10–30 days				0.10
Yes	20/109	18.3	1.51 (0.92, 2.46)	
No	621/4,781	13.0	1	
Living in a residence with a smoker				0.36
Yes	250/1,823	13.7	1.08 (0.91, 1.28)	
No	395/3,087	12.8	1	
Parent/guardian working in medical care setting				0.39
Yes	40/269	14.9	1.16 (0.82, 1.65)	
No	605/4,637	13.0	1	
Living with siblings				<0.01
Yes	345/1,722	20.0	2.42 (2.05, 2.87)	
No	298/3,179	9.4	1	
Attending day-care				<0.01
Yes	140/440	31.8	3.67 (2.94, 4.57)	
No	505/4,471	11.3	1	
Prior pneumococcal vaccine				<0.01
Received pneumococcal vaccine	260/2,342	11.1	0.71 (0.60, 0.84)	
Received PCV (PCV7 or PCV13) only	232/2,162	10.7	0.68 (0.57, 0.81)	
Received PPSV23	28/180	15.6	1.10 (0.72, 1.67)	
Without pneumococcal vaccine	367/2,458	14.9	1	

CI, confidence interval; OR, odds ratio; PCV, pneumococcal conjugate vaccine; PPSV, pneumococcal polysaccharide vaccine.

no. = total number of *S. pneumoniae* carriage within the specified category.

n = total number of subjects included in the specified category.

^a^ 95% CI was calculated with the Wald method (i.e., asymptotic normality).

In Beijing, the strict administration of COVID-19 prevention and control measures began in January 2020. By study year, the highest *S. pneumoniae* carriage prevalence was found in 2019 (15.8%), in both study centers, and the lowest carriage prevalence occurred in 2020 (10.6%) (*P* < 0.01, Table 1). The carriage rate of *S. pneumoniae* in older children (25 to <60 months) in Beijing decreased from 22.2% (53/239) in 2018–2019 to 5.8% (32/557) in 2020–2021 (*P* < 0.01), which had a more marked change than in Shenzhen where the carriage rates were 22.0% in 2018–2019 and 21.4% in 2020–2021 (Supplementary Table S2).

The *S. pneumoniae* carriage rate was 20.0% in children living with siblings vs. 9.4% without siblings, OR: 2.42 [95% CI: 2.05–2.87]. The carriage rate of *S. pneumoniae* in children attending day-care was 31.8% vs. 11.3% for those not attending day care centers, OR: 3.67 [95% CI: 2.94—4.57. *S. pneumoniae* carriage prevalence associated with the history of PCV7 or PCV13 vaccination, which was 14.9% in children without pneumococcal vaccine, 11.4% in those who received 1, 2, or 3 doses of PCV7 or PCV13, and 9.4% in those children who received 4 doses of PCV13 (Supplementary Figure S2), while there was no effect of PPSV23 (15.6%). Other tested predictors of *S. pneumoniae* carriage were not statistically significant, including gender, receiving an antimicrobial within the past 10–30 days, living in a residence with a smoker, or parent/guardian working in a medical institution.

### 3.3 Serotype distribution

**Figure 1:**
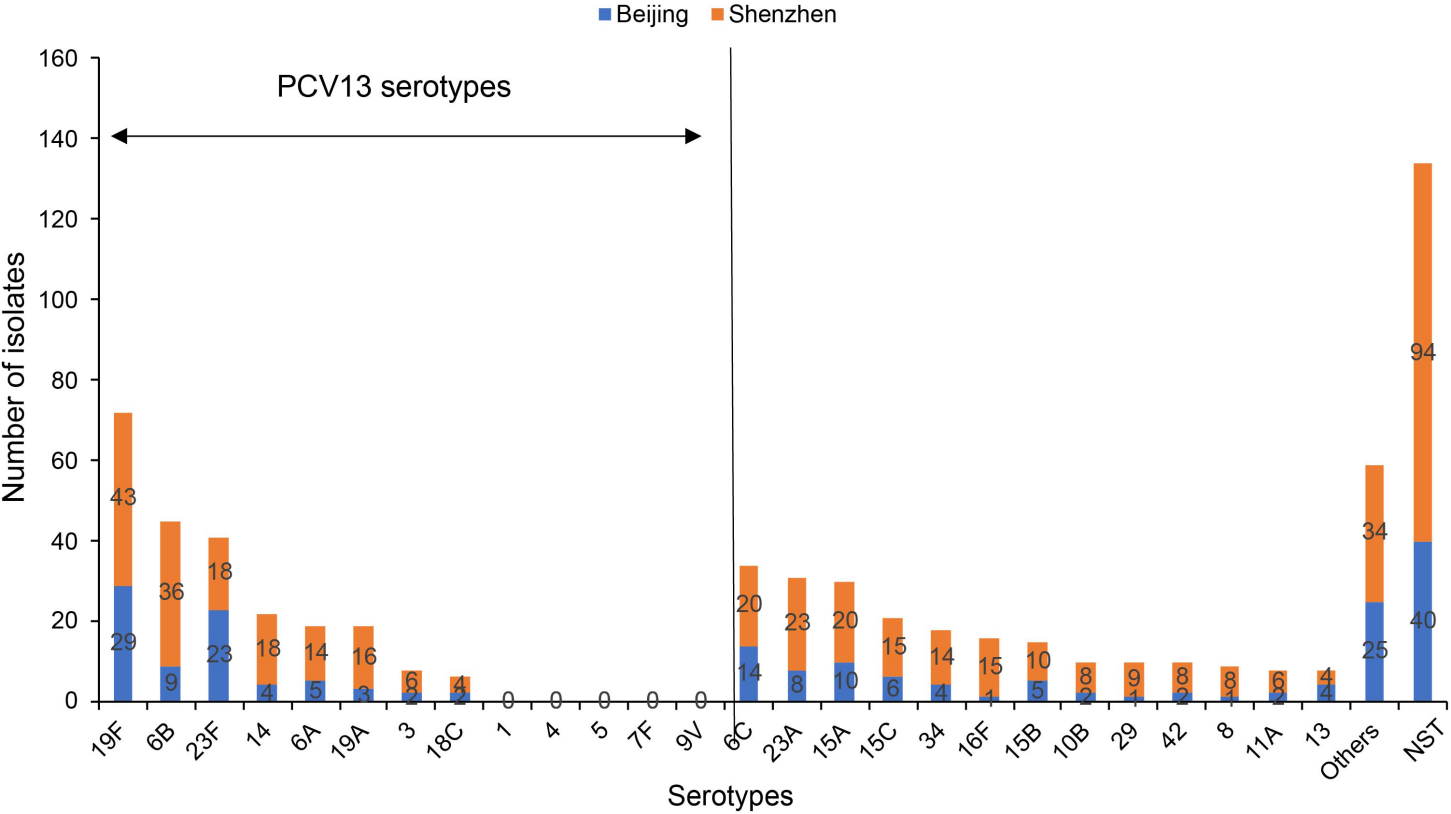
The most common pneumococcal serotypes identified in nasopharyngeal swabs collected from Chinese children aged 30 days to <60 months, shown by region (Beijing and Shenzhen). NST, non-serotypeable *S. pneumoniae*; PCV, pneumococcal conjugate vaccine. Others includes four isolates each for type 20,6D and 7C; three isolates each for type 9V, 10A,17F, 23B and 24F; two isolates each for type 1, 9N, 7A, 9A, 11B, 11C, 11F, 18B, 28F and 40; one isolate each for type 22F, 33F, 7B, 16A, 19B, 19C, 24B, 28A, 35F, 36, 37 and 43.

In this study, there were 645 *S. pneumoniae* isolates obtained from all participants included in the study, and among the 511 encapsulated *S. pneumoniae* isolates, 51 different serotypes were identified. Among all *S. pneumoniae* isolates, the most frequent serotypes were 19F (72/645, 11.2%), 6B (45/645, 7.0%), 23F (41/645, 6.4%), and 6C (34/645, 5.3%). The spectrum of carriage serotypes differed between Beijing and Shenzhen (Figure 1). Among isolates collected in Beijing, the most frequent serotypes were 19F (29/202, 14.4%), 23F (23/202, 11.4%), 6C (14/202, 6.9%), and 15A (10/202, 5.0%). Among isolates collected in Shenzhen, most frequent serotypes were 19F (43/443, 9.7%), 6B (36/443, 8.1%), 23A (23/443, 5.2%), 6C (20/443, 4.5%), and 15A (20/443, 4.5%).

**Figure 2:**
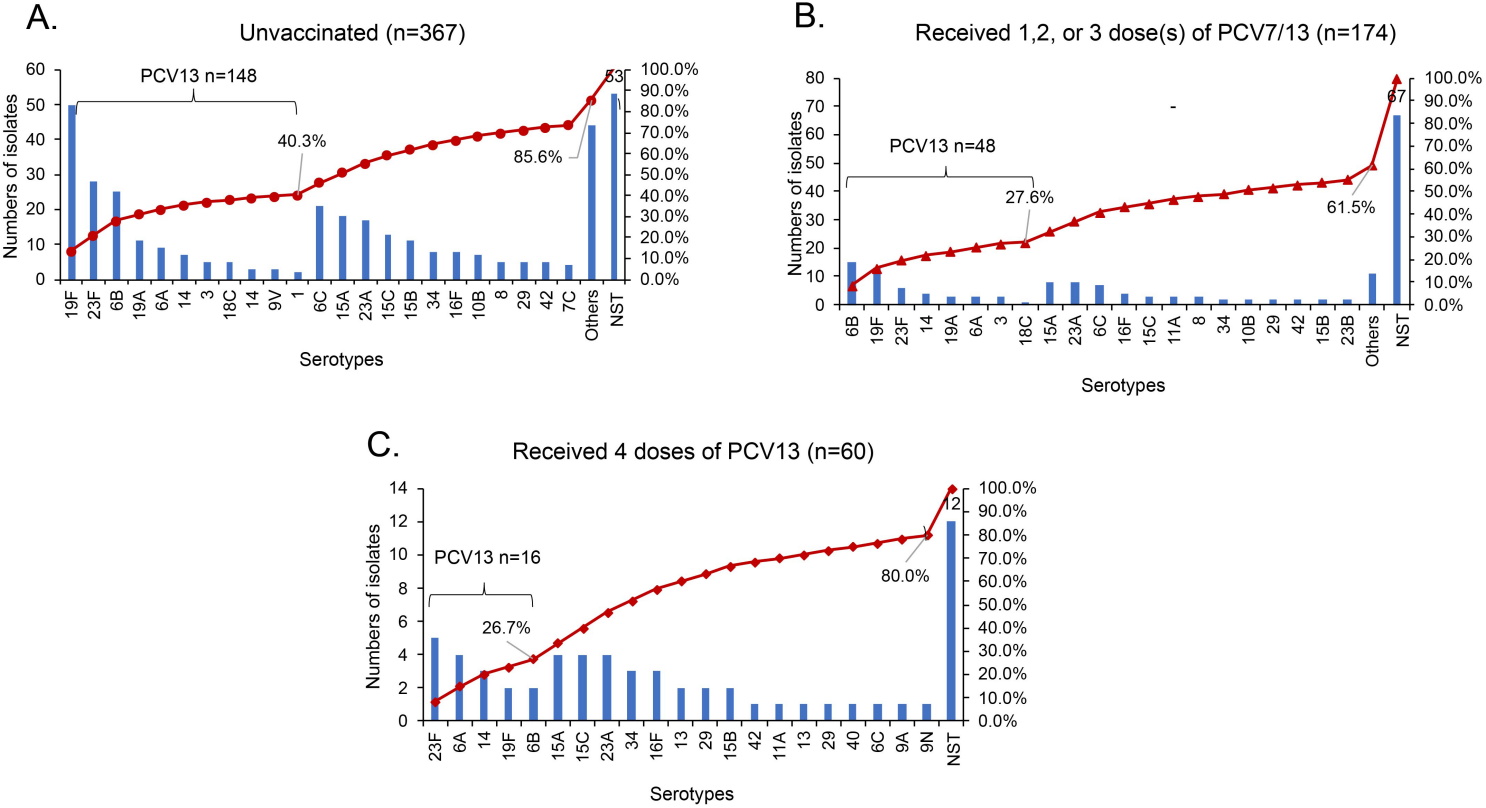
The serotype distribution of *S. pneumoniae* in nasopharynx of children aged 30 days to <60 months with different vaccination status. NST, non-serotypeable *S. pneumoniae;* PCV: pneumococcal conjugate vaccine. **(A)** Other includes three isolates each for type 11A, 17F, 20, 24F, 34 and 6D; two isolates each for type 10A, 11C, 11F, 13 and 42; one isolate each for type 11B, 16A, 18B, 19B, 19C, 22F, 24B, 28A, 28F, 7A, 7B, 9A, 13, 36, 37 and 43. **(B)** Other includes one isolate each for type 10A, 11B, 18B, 28F, 33F, 35F, 6D, 7A, 9N, 13 and 40.

Among the 645 *S. pneumoniae* isolates (511 encapsulated and 134 non-serotypeable), the estimated serotype coverage for PCV7, PCV13, PCV15, PCV20, and PPSV23 was 29.3% (*n* = 189), 36.7% (*n* = 237), 37.1% (*n* = 239), 42.5% (*n* = 274), and 40.9% (*n* = 264), respectively. The serotype distribution and pneumococcal vaccine coverage are shown according to age group in Supplementary Figure S3. The vaccine coverage increased by age group. By contrast, the non-PCV13 serotypes coverage rates decreased significantly from 74.4% for 30 days to <6 months, 66.7% for 7–12 months, 64.7% for 13–24 months, and 54.7% for 25 to <60 months (*χ*^2^ = 12.448, *P* = 0.006). The most frequent non-PCV13 serotypes included: 6C, 23A, 15A and 15C. However, non-PCV13 serotype order differed slightly with PCV13 history: the top non-PCV13 serotypes were 15C, 15A and 23A among those fully vaccinated with PCV13; 15A, 23A and 6C among those partially vaccinated with PCV13; and 6C, 15A and 23A among those receiving no PCV13 (Figure 2).

The serotype distribution of *S. pneumoniae* in the nasopharynx of children aged 30 days to 60 months by vaccination status is shown in Figure 2. In unvaccinated children, PCV13 serotypes accounted for 40.3% of the total serotypes overall, and the most prevalent serotypes were 19F, 23F, and 6B (Figure 2A). Among the 174 children vaccinated with ≤3 doses of PCV13, PCV13 serotypes account for 27.6% of all isolates, and serotype 6B (8.6%) accounted for the highest proportion, followed by 19F (7.5%) and 23F (3.4%). In this group of 174 children vaccinated with ≤3 doses of PCV13, only 61.5% of the *S. pneumoniae* isolates were serotypeable *S. pneumoniae* (Figure 2B). Among the 60 children who received the 4-dose PCV13 series, the PCV13 serotypes accounted for 26.7% of all serotypes. The most common PCV13 serotypes included 23F (8.3%) and 6A (6.7%), and the predominant non-PCV13 serotypes were 15A (6.7%), 15C (6.7%), and 23A (6.7%). The serotypeable *S. pneumoniae* proportion was 80.0% (Figure 2C).

### 3.4 Antimicrobial susceptibility

The susceptibility pattern and MIC distribution to 10 antimicrobials of 644 *S. pneumoniae* isolates (1 strain isolated in Beijing failed to survive for drug susceptibility testing) are listed in Table 2. Most isolates were susceptible to amoxicillin (93.5%), penicillin (non-meningitis) (92.1%), ceftriaxone (88.8%), imipenem (75.0%), meropenem (68.5%), levofloxacin (98.9%), or linezolid (99.2%). Furthermore, all isolates evaluated were susceptible to vancomycin (100%). In contrast, an elevated proportion of resistant isolates was observed for penicillin (meningitis) (72.4%), erythromycin (96.3%), and trimethoprim/sulfamethoxazole (68.2%).

**Table 2:** Antimicrobial susceptibility test results of 644 isolates of *S. pneumoniae* isolated from Chinese children aged 30 days to <60 months, 2018–2021.

Antimicrobials	MIC (mg/L)			Susceptibility (%)^b^			
	Class	MIC_50_	MIC_90_	Range	R	I	S
Amoxicillin	I	0.25	2	0.008–512	4.3	2.2	93.5
Penicillin (non-meningitis)	I	0.25	2	0.008–64	3.7	4.2	92.1
Penicillin (meningitis)	I	0.25	2	0.008–64	72.4	5.1	22.5
Penicillin (oral)	I	0.25	2	0.008–64	20.7	56.8	22.5
Ceftriaxone^a^	II	0.25	2	0.008–512	4.0	7.1	88.8
Imipenem	IV	0.0625	0.25	0.004–64	2.0	23.0	75.0
Meropenem	IV	0.125	1	0.004–128	10.4	21.1	68.5
Erythromycin	III	512	512	0.094–512	96.3	0.3	3.4
Levofloxacin	V	0.5	1	0.125–64	0.5	0.6	98.9
Linezolid	VI	1	2	0.25–4	0.8	0	99.2
Vancomycin	VI	0.5	0.5	0.125–1	0	0	100.0
Trimethoprim/sulfamethoxazole	VII	16	2048	0.016–2048	68.2	11.5	20.3

I, intermediate; MIC, minimum inhibitory concentration; PCV, pneumococcal conjugate vaccine; R, resistant; S, sensitive.

^a^ Ceftriaxone is interpreted according to non-meningitis.

^b^ If the number of *S. pneumoniae* isolates tested for antibiotic sensitivity is less than the total number of *S. pneumoniae* isolates, the actual number of detected strains is used as the denominator in this table.

From a total of 158/644 isolates (24.5%), 21 serotypes (included 3, 6A, 6B, 6C, 6D, 7C, 8, 10B, 11A, 14, 15A, 15B, 15C, 16F, 18B, 19A, 19F, 23A, 23F, 24F, 34) were MDR. For the PCV13 covered isolates, MDR was detected in 36.3% (86/237) of isolates; in comparison, 17.1% (25/274) of non-PCV13 serotypes, including NST isolates, were MDR. In particular, PCV13 serotypes had a statistically significantly higher resistance rates to penicillin (meningitis) (87.3% vs. 66.3%; *P* < 0.01), penicillin (oral) (31.6% vs. 14.3%; *P* < 0.01), and meropenem (20.7% vs. 4.4%; *P* < 0.01) (Table 3). For PCV15, PCV20, and PPSV23 covered isolates, MDR was detected in 87/239 isolates (36.4%), 98/274 isolates (35.8%), and 91/264 isolates (34.5%), respectively.

**Table 3:** Comparison of resistance rates between PCV13 and non-PCV13 isolates in Beijing and Shenzhen.

Antimicrobial	PCV13 *n* = 237 (%)	Non-PCV13 serotypes		
		Encapsulated *S. pneumoniae n* = 274 (%)	NST *n* = 133^b^ (%)	*P* value
MDR	86 (36.3)	25 (9.1)	47 (35.3)	<0.01
Amoxicillin	3 (1.3)	23 (8.4)	2 (1.5)	<0.01
Penicillin (non-meningitis)	4 (1.7)	18 (6.6)	2 (1.5)	<0.01
Penicillin (meningitis)	207 (87.3)	157 (57.3)	102 (76.7)	<0.01
Penicillin (oral)	75 (31.6)	55 (20.1)	3 (2.3)	<0.01
Ceftriaxone^a^	6 (2.5)	18 (6.6)	2 (1.5)	0.09
Erythromycin	230 (97.0)	265 (96.7)	125 (94.0)	<0.01
Imipenem	5 (2.1)	5 (1.8)	3 (2.3)	0.949
Meropenem	49 (20.7)	14 (5.1)	4 (3.0)	<0.01
Levofloxacin	0 (0)	1 (0.4)	2 (1.5)	0.097
Linezolid	3 (1.3)	2 (0.7)	0 (0)	0.477
Vancomycin	0 (0)	0 (0)	0 (0)	–
Trimethoprim/sulfamethoxazole	172 (72.6)	215 (78.5)	52 (39.1)	<0.01

MDR, multi-drug resistant; NST, non-serotypeable *S. pneumoniae;* PCV, pneumococcal conjugate vaccine.

^a^ Ceftriaxone is interpreted according to non-meningitis.

^b^ There was one strain (NST) isolated in Beijing that failed to survive after storage for the drug susceptibilities test.

## 4 Discussion

In this study conducted among Chinese children 30 days to <60 months of age*,* overall *S. pneumoniae* NP carriage was 13.1%. These results were similar to the carriage prevalence reported from children younger than 5 years in Taiwan (12.0%, 60/500) (21), but lower than the NP carriage observed from other studies in Ghana (32.6%, 63/193) (22), Ethiopia (43.8%, 311/710) (23), or Turkey (17.8%, 103/580) (24). In a previous meta-analysis performed in China prior to the introduction of PCV7 that included children younger than 5 years of age, NP carriage was 24.4% (25). In an earlier study conducted between 2012 and 2014 by the Beijing Center for Disease Control and Prevention (CDC) among unvaccinated children 2–5 years old, it was reported that among 3,281 children aged 3.1 (±0.8) years, pneumococcal NP carriage was detected in 22% of the samples (25, 26). Potential explanations for the lower NP pneumococcal carriage observed among Chinese children in the current study could be related to: the increasing uptake in PCV13 among Chinese children (27); the fact that the study was conducted during COVID-19 pandemic with all the implemented recommendations in China to prevent viral respiratory transmission (28, 29); and the low number of children per family and the custom that children under 3 years of age are cared for at home generally by grandparents or nannies instead of at day care centers, as the entry for public day nursery is 3 years of age (30, 31).

We found that vaccination with PCV13 reduced pneumococcal carriage. While an indirect protection with PCV13 against PCV13 serotypes is well-established when PCV13 is included in the national immunization program, the results of the current study also suggest that even in the setting of private market uptake, there may have been an indirect benefit of carriage. As reported previously (32), we found that vaccination with PPSV23 did not influence NP carriage, reflecting an inherent limitation of plain polysaccharide vaccine.

Dominant serotypes differed slightly with PCV13 vaccination history: the top serotypes among unvaccinated children were 19F, 23F, 6B, and 6C, whereas serotypes 15A, 6B, 19F, 15A, 23A, and 6C were the most frequent serotypes among children partially vaccinated with PCV7 (3 children) or PCV13; and 23F, 6A, 15C, 15A, and 23A were the most frequent serotypes observed among children fully vaccinated with PCV13. The association between PCV vaccination and lower vaccine serotype NP carriage has been previously described (33). In Greece, for instance, where PCVs have been in the NIP since 2006, initially with PCV7 and since 2010 with PCV13 at an estimated four-dose uptake rate of about 80%, molecular surveillance of pneumococcal carriage following completion of immunization with the PCV13 (3 + 1 schedule) revealed that non-PCV13 serotypes represented 83.8% of total isolates. Serotypes 19A and 3 were the only two PCV13 serotypes that increased in proportion over the time of the study (*P* < 0.001 and *P* = 0.012, respectively) (33).

The percentage of non-serotypeable *S. pneumoniae* (20.7%) isolates observed in our study is consistent with results from studies conducted in other geographical locations (34–36), which helps to support the representativeness of the samples and the validity of the research results.

In the present study, children enrolled in Beijing had lower *S. pneumoniae* NP carriage compared with children enrolled in Shenzhen, which became most apparent in the 2020–2021 COVID-19 period. As noted, the carriage rate of *S. pneumoniae* in Beijing decreased significantly in the post-COVID period, but the carriage rate in Shenzhen did not. The decreasing carriage rate of *S. pneumoniae* in children in Beijing during the COVID-19 pandemic could be related to the continuous and strict prevention and control measures (such as universal masks, distancing, and hand washing). For instance, in Beijing closed kindergartens from February 2020 until the end of this study while Shenzhen did not. These measures contributed to limiting the transmission of COVID-19, but also reduced the spread of other pathogens. From January 2020 to February 2020, the spread of the epidemic in China had been first curbed. Most of the declared infectious disease incidences showed a downward trend in 2020 in China (37). Other settings outside of China did not observe such a COVID-19 pandemic effect. For instance, in a prospective cohort study in Israel, the mean proportion of children younger than 3 years old carrying pneumococcus, which was 44.3 ± 1.9% during 2016–2019, was somewhat reduced in October–December 2020, although the rates during January–February 2021 were not significantly different from those in the pre-COVID period (38). Nonetheless, unexamined interregional factors (e.g., climate, population characteristics, and economic levels) might have contributed to the observed differences in colonization in China during the COVID-19 pandemic and modified *S. pneumoniae* transmission.

To our knowledge, this present study (2018–2021) is the largest pneumococcal carriage study conducted in healthy children in China. Serotype 19F (11.2%) was the most prevalent *S. pneumoniae* carriage serotype, followed by 6B (7.0%), 23F (6.4%), 6C (5.3%), and 23A (4.8%). A meta-analysis among Chinese children in the post-PCV7 era (from October 2008 to 2016) reported that serotypes 19F, 6A, and 23F were most often isolated (24). A systematic review and meta-analysis of studies of carriage of *S. pneumoniae* and other respiratory bacterial pathogens in LMICs reported that serotypes 6A, 6B, 19A, 19F, and 23F were the serotypes most often isolated (35). In our study, the top five circulating serotypes included two non-PCV13 serotypes (6C and 23A). Researchers from Germany (39), Canada (40), Gambia (41) and Israel (42) suggested that increases in the carriage of non-PCV13 serotypes had offset reductions in the carriage prevalence of vaccine-targeted serotypes. Furthermore, the present study focuses on the children living in only two Chinese cities, where 47.7% of children had received at least one dose of PCV13. The present results demonstrated that 63.3% (408/645) of isolates obtained were non-PCV13 serotypes (e.g., 6C, 23A, 15A, 15C, 34, 16F, and 15B). Between 2014 and 2019, Xu et al. (43) reported that 12.5% (2/16) of fatal IPDs were caused by non-PCV13 serotypes (15A and 15B) that were not identiﬁed before the introduction of PCV13. Therefore, it is particularly important to monitor the serotype of *S. pneumoniae* in the nasopharynx of healthy children.

The highest antimicrobial resistance rates were observed for erythromycin (96.6%) or trimethoprim/sulfamethoxazole (68.2%), while resistance to penicillin (based on the breakpoints for non-meningitis isolates) and other beta-lactam agents was low (<5%). The MIC50 and MIC90 against erythromycin was >256 mg/L, which could reflect macrolide overuse in China (44). The high macrolide resistance is consistent with data reported in other Asian countries (45).

A total of 158 (24.5%) isolates were MDR. Vaccine-serotype pneumococcal isolates possessed more MDR than non-vaccine serotypes, which emphasizes the role for PCV immunization in the control of pneumococcal disease associated with antibiotic resistance. National vaccination using PCVs prevent episodes of both *S. pneumoniae* diseases and carriage, the latter which can inhibit the spread of antibiotic resistance (46). As non-PCV13 serotypes such as 11A and 24F are associated with antimicrobial resistance, further surveillance is needed (47). Of note, the MDR isolates of non-vaccine serotypes in this study increased year by year during the study period. We found that the rate of MDR non-PCV13 serotypes (including NST) in 2018 was 0.0%, followed by an increase towards 9.6% in 2019, 18.1% in 2020, and 18.7% in 2021. These MDR non-PCV13 strains can evade, at the same time, the pressures of vaccine-induced immunity and antimicrobial selection. The potential relationship between antimicrobial selective pressure and serotype replacement is a reminder that antimicrobials should be used properly to ensure that PCVs remains a powerful tool in the fight against antimicrobial resistance in *S. pneumoniae*.

One limitation of the present study is that the study was conducted in only in two cities with strong economies and wide availability of medical care, which does not reflect the diversity of China. Further investigations could provide more information about *S. pneumoniae* NP carriage. Other limitations of this study were a relatively modest pre-NIPs sample size. To compensate, we drew upon the pneumococcal NP swab carriage study conducted previous as an additional reference point (25, 26).

This investigation revealed that one-seventh or one-eighth of children <60 months of age carried *S. pneumoniae* in their nasopharynx in Beijing and Shenzhen, and like previous studies, *S. pneumoniae* carriage prevalence increased for subjects of older age (25 to <60 months), those living with siblings, attending day nursery, or who were unvaccinated with PCV13. The strict administration of measures to control COVID-19 led to a decrease in *S. pneumoniae* carriage rates, which was particularly evident in Beijing. Although PCV immunization uptake was limited, it showed a preventive effect (individual protection) on vaccine serotype *S. pneumoniae* carriage among vaccinated healthy young children in the community, providing evidence that control of *S. pneumoniae* carriage through vaccination could have a positive effect on antibiotic resistance.

## Data Availability

The original data contributions presented in the study are included in the article or supplementary material. Further inquiries can be directed to the corresponding author(s).
