# Supplementary material for: Nasopharyngeal carriage of Streptococcus pneumoniae among children aged 30 days to <60 months in Beijing and Shenzhen, China (2018–2021) during pneumococcal conjugate vaccine introduction and the coronavirus disease (COVID-19) pandemic
Source: Front Pediatr. 2024 Sep 3;12:1382165. doi: 10.3389/fped.2024.1382165 (PMC11421034; doi:10.3389/fped.2024.1382165)
Supplement: Supplementary file 1 [file Table1.docx]

| **Supplementary Table 1. Vaccine information of the enrolled healthy children aged 30 days to < 60 months in Beijing and Shenzhen** | | | | |
| --- | --- | --- | --- | --- |
|  | **Beijing(N=2435)**  **n (%)** | | **Shenzhen(N=2476)**  **n (%)** | **Total(N=4911)**  **n (%)** |
|  | | | | |
| Received pneumococcal vaccine | 988 (40.6%) | | 1354 (54.7%) | 2342 (47.7%) |
| 3-dose PCV7 | | 3 (0.1%) | 0 | 3 (0.1%) |
| 1-dose PCV13 | | 105 (4.3%) | 208 (8.4%) | 313 (6.4%) |
| 2-dose PCV13 | | 98 (4.0%) | 208 (8.4%) | 306 (6.2%) |
| 3-dose PCV13 | | 396 (16.3%) | 505 (20.4%) | 901 (18.3%) |
| 4-dose PCV13 | | 322 (13.2%) | 317 (12.8%) | 639 (13.0%) |
| 1-dose PPSV23 | | 63 (2.6%) | 103 (4.2%) | 166 (3.4%) |
| Mixed PCV13/PPSV23 | | 1 (0.0%) | 13 (0.5%) | 14 (0.3%) |
| No pneumococcal vaccine received | | 1365 (56.1%) | 1093 (44.1%) | 2458 (50.1%) |
| Uncertain ^a^ | | 82 (3.4%) | 29 (1.2%) | 1. (2.3%) |

1. Uncertain vaccination information was defined as those who were failure to present the vaccination booklet and did not have a clear vaccination record in the registration network.

**Supplementary Table 2. Pneumococcal carriage in different age group in Beijing /Shenzhen before and after the COVID-19 pandemic**

|  | Beijing | | Shenzhen | |
| --- | --- | --- | --- | --- |
|  | 30days-24months | 25-<60 months | 30days- 24months | 25-<60months |
| 2018-2019 | 11.78% | 22.18% | 9.82% | 22.02% |
| 2020-2021 | 5.89% | 5.75% | 17.92% | 21.43% |
| χ2 | 14.368 | 47.336 | 15.158 | 0.025 |
| *P* | 0.001 | 0.001 | 0.001 | 0.874 |
