# Supplementary figures and images for: Nasopharyngeal carriage of Streptococcus pneumoniae among children aged 30 days to <60 months in Beijing and Shenzhen, China (2018–2021) during pneumococcal conjugate vaccine introduction and the coronavirus disease (COVID-19) pandemic
Source: Front Pediatr. 2024 Sep 3;12:1382165. doi: 10.3389/fped.2024.1382165 (PMC11421034; doi:10.3389/fped.2024.1382165)

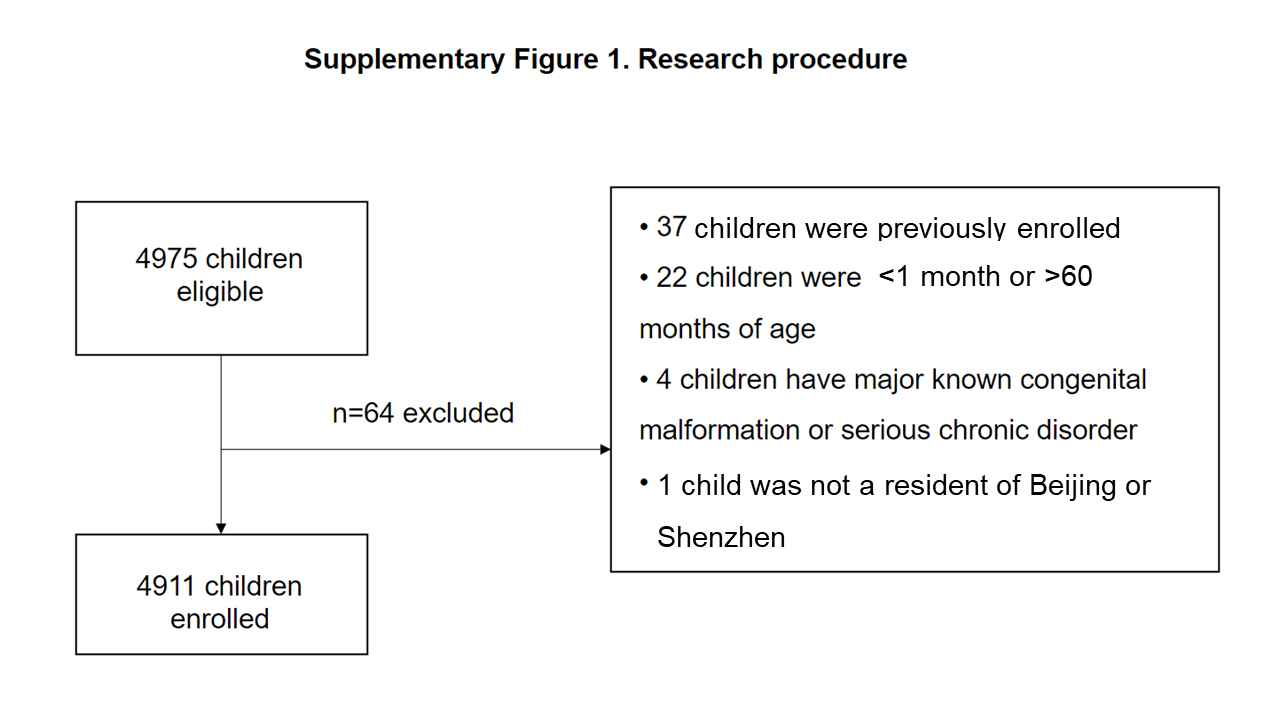

Supplement: Supplementary file 3 [file Image1.tif]

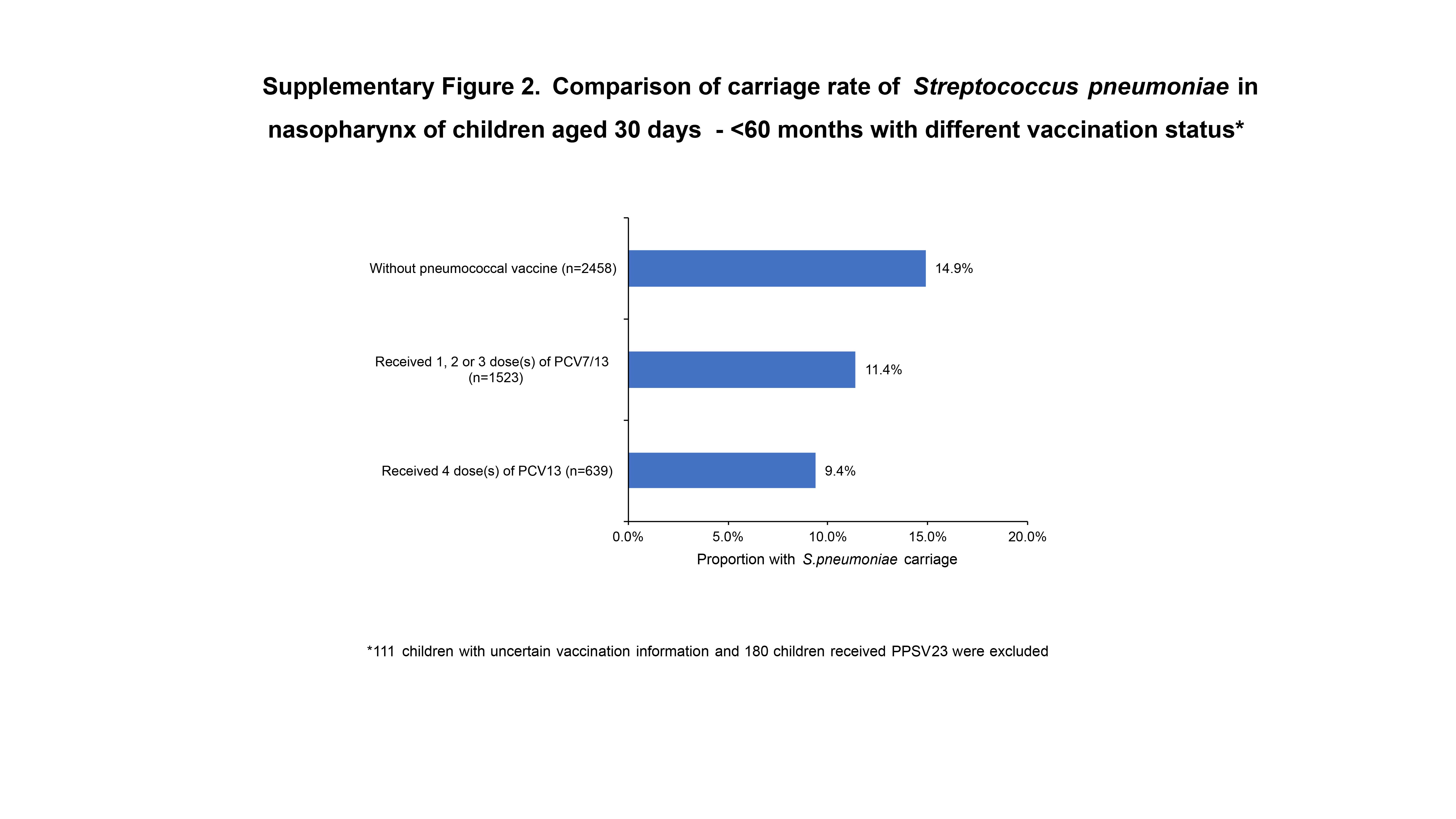

Supplement: Supplementary file 4 [file Image2.jpeg]

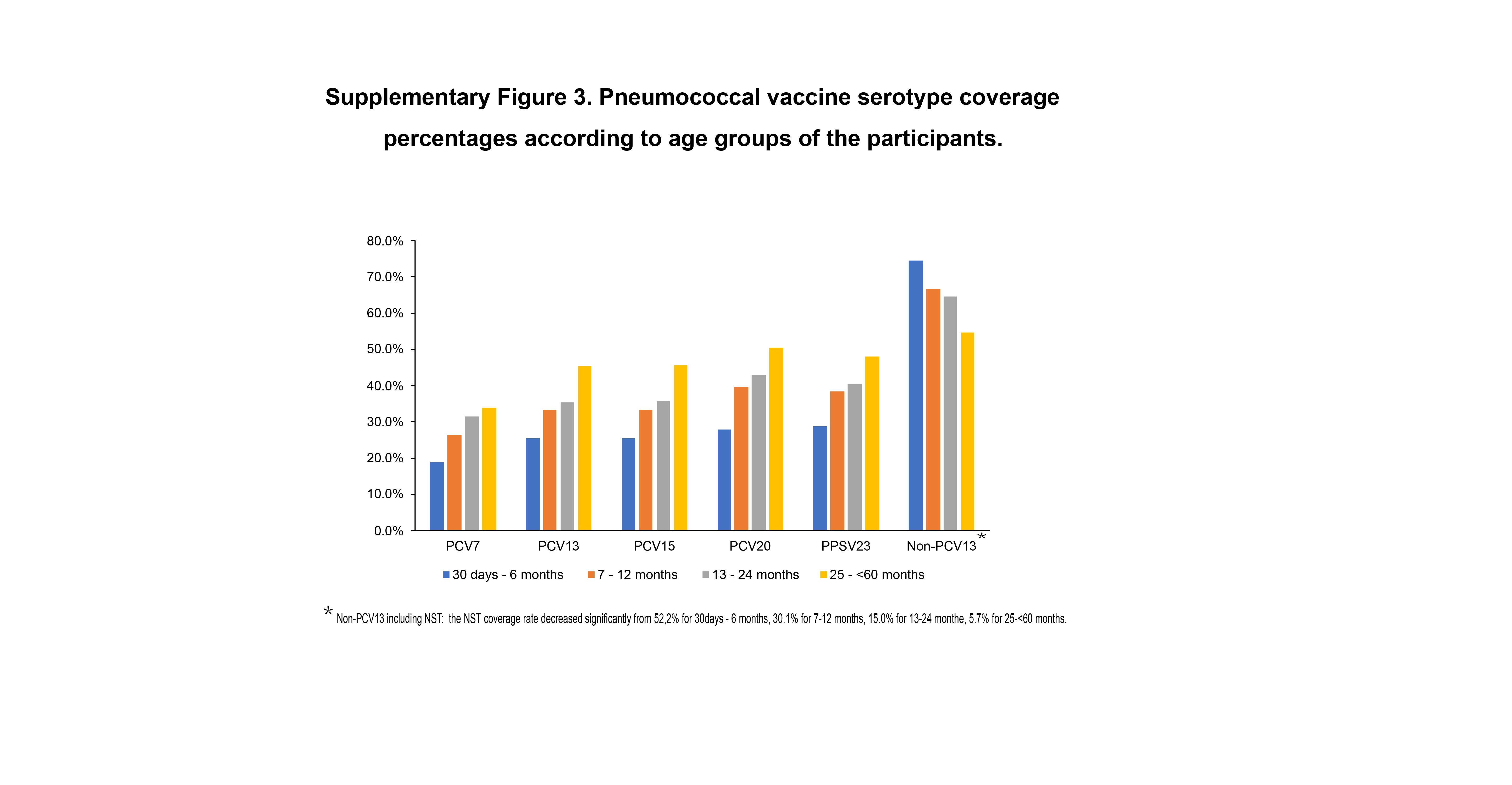

Supplement: Supplementary file 5 [file Image3.jpeg]
